# Pseudotumor Deltoideus: Touch Me Not Lesion of the Humerus

**DOI:** 10.7759/cureus.37427

**Published:** 2023-04-11

**Authors:** Michael Chirayath, Stallon Sebastian, Arun George

**Affiliations:** 1 Radiology, St. John's Medical College Hospital, Bangalore, IND

**Keywords:** humerus lucency, t2 isointense, t1 isointense, benign, pseudotumor deltoideus, deltoid lucency, cortical thickening

## Abstract

Pseudotumor deltoideus is a localized region of irregular cortical thickening at the deltoid insertion that causes a diagnostic quandary for radiologists due to its unusual radiological characteristics. It is benign in origin, with the potential to act as a tumor stimulator, and has a variety of anatomic variants. The lesions can be characterized on an X-ray by an area of lucency at or around the deltoid tuberosity and a cortical irregularity or nearby eccentric marrow abnormality on computed tomography (CT)/magnetic resonance imaging (MRI). The presence of cortical thickening and lucency at the deltoid insertion are unusual radiological findings that provide a diagnostic challenge. In this article, we provide cases of shoulder pain together with radiological imaging to make this previously underappreciated condition more understandable. Further evaluation with CT/MRI should be performed in all cases of shoulder pain with conventional radiographic findings of cortical thickening and intracortical lucency. The diagnosis of the condition is aided by the presence of elongated lucency on CT and T2 hyperintensity in the cortex of the proximal humerus. The clinical and imaging characteristics are important in the diagnosis of this condition. It must not be confused for infection or malignancy, and a biopsy must never be attempted.

## Introduction

Morgan et al. first used the term “pseudotumor deltoideus” in 2001 after they published a study on individuals who had shoulder pain and observed similar radiological clues at the deltoid insertion [1,2]. It is well-recognized that deltoid muscle insertion is the site of a few benign skeletal anatomical abnormalities that appear as irregular cortical thickening [1]. Similar cortical irregularity and thickening at the deltoid insertion were also brought on by chronic avulsion damage to the deltoid tubercle and the pectoralis major insertion in the proximal humerus. As a result, the presence of such skeletal abnormalities, combined with pain and impairment, can mimic some cancers or infections. Therefore, these lesions are most often misdiagnosed and subjected to unnecessary investigations [1]. This article illustrates three cases and aims to better understand the radiological clues of this unusual variant known as pseudotumor deltoideus, a lesser-known cause of shoulder pain.

## Case presentation

Case I

A 54-year-old male presented with a complaint of chronic left shoulder pain with mild restriction of movements. There was no history of trauma, significant weightlifting, or sports participation. On examination, there was pain associated with abduction and internal rotation of the nondominant left shoulder. Imaging revealed irregular cortical thickening, intracortical lucencies, and hyperintense T2-weighted lesion at the site of the deltoid muscle insertion. Based on these clinical and radiological findings, the diagnosis of pseudotumor deltoideus was made, and conservative treatment was advised. The patient was started on nonsteroidal anti-inflammatory drugs (NSAIDs), as well as regular physiotherapy and hot fomentation, and was then called for a follow-up appointment. The severity of the shoulder pain had significantly decreased at the two-month follow-up evaluation, and the complete range of motion had been restored. The follow-up X-ray, however, revealed no change. 

**Figure 1:**
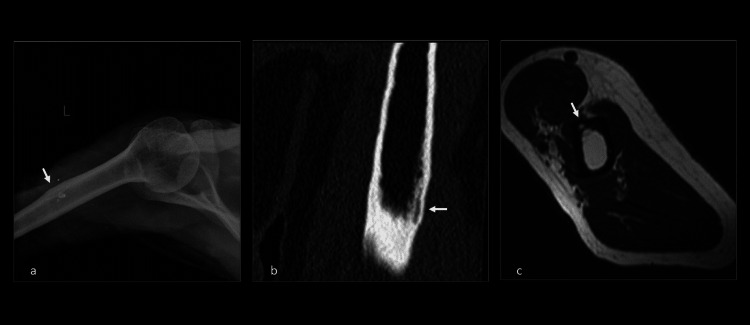
X-ray, CT, and MRI evaluation of the left shoulder. (a) Frontal radiograph of the left shoulder shows an ill-defined area of cortical thickening and sclerosis at the deltoid insertion. (b) Coronal CT of the left humerus shows well-defined cortical thickness, mild irregularity, and intracortical lucencies along the endosteal cortex. (c) Axial T2WI shows small hyperintense foci in the endosteal region along the anteromedial aspect of the humerus at the insertional site of the deltoid. CT, computed tomography; MRI, magnetic resonance imaging; T2WI, T2-weighted image

Case II

A 39-year-old female presented to OPD with a six-week history of left shoulder pain and difficulty with overhead movement. On physical examination, there was a mild restriction of overhead abduction. She was right-hand dominant with no associated history of trauma. Imaging revealed similar irregular cortical thickening and intracortical lucent lesion. She was managed conservatively and showed significant symptom resolution.

**Figure 2:**
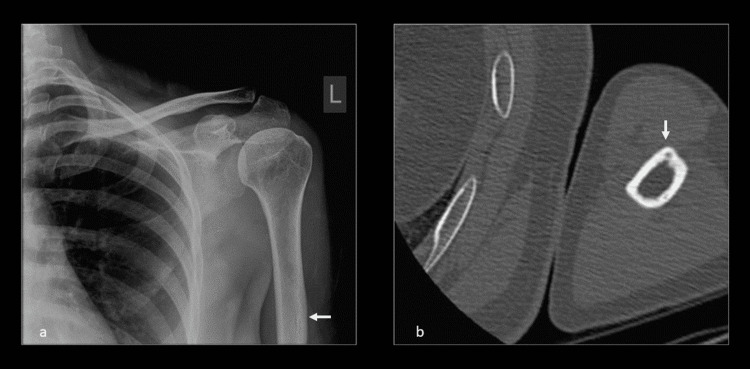
X-ray and CT evaluation of the left shoulder. (a) Frontal radiograph of the left humerus shows endosteal and cortical thickening laterally at the level of the deltoid insertion. (b) Axial CT of the left humerus shows thickened lateral cortex and radiolucent center of the lesion. CT, computed tomography

Case III

A 27-year-old right-hand-dominant female presented with a long-standing history of right upper and mid-arm pain. There was no associated history of trauma or sports-related injury. The physical examination of the patient revealed pain with internal and external shoulder rotation. Computed tomography (CT) and magnetic resonance imaging (MRI) evaluation revealed an intracortical lucent lesion with T1 hypointense and T2 hyperintense signal changes suggestive of pseudotumor deltoideus. He was managed conservatively, and the follow-up showed a resolution of symptoms.

**Figure 3:**
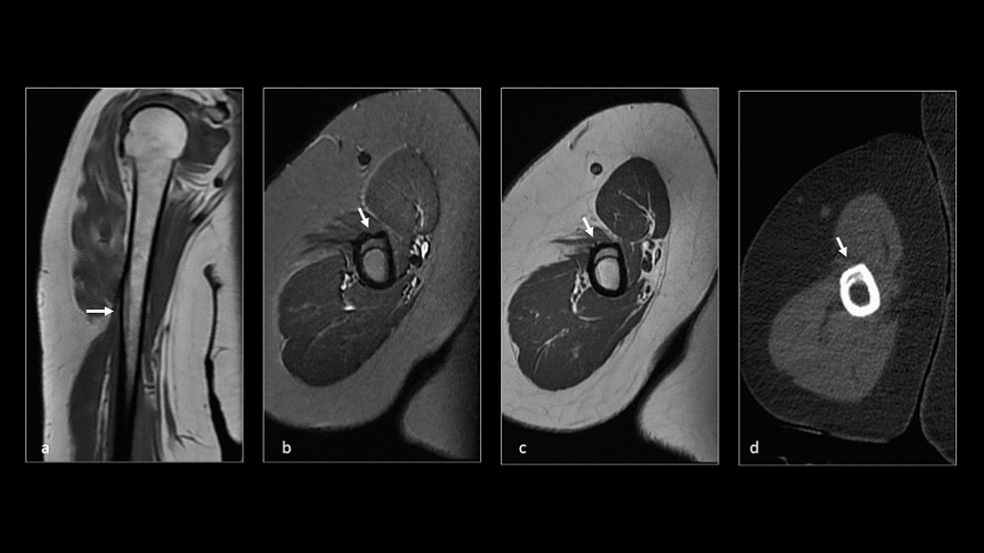
CT and MRI evaluation of the right shoulder. (a) Coronal T1WI shows an isointense area of cortical thickening. (b and c) Axial T2 STIR and T1WI hyperintense area of cortical thickening compared to the normal cortical bone signal intensity at the region of the deltoid insertion in the right humerus with no evidence of adjacent marrow and soft tissue edema. (d) Axial CT shows well-defined cortical thickening, expansion, and mild irregularity on the endosteal surface with irregular intracortical lucency at the deltoid muscle attachment area. CT, computed tomography; MRI, magnetic resonance imaging; T1WI, T1-weighted image; T2 STIR, T2-weighted short-tau inversion recovery

## Discussion

When an individual with a history of shoulder pain is observed in conjunction with imaging features such as irregular cortical thickening and intracortical lucency in the proximal humerus, it can lead to an unneeded biopsy or investigations based on the assumption of a malignant disease [1-3]. To add to the confusion further, both musculoskeletal malignancy and sports-related injuries affect young patients, and their symptoms frequently overlap [4]. However, imaging investigations of these lesions revealed no underlying malignancy, and a clinical follow-up indicates that the process is inactive. Hence, “pseudotumor deltoideus” is a term used to describe an anatomical variant at the deltoid insertion.

Although several theories have been proposed to explain this phenomenon, it is uncertain what really caused it. A few possible explanations include an anatomic variant, erosions associated with tendinitis, secondary changes at the deltoid insertion due to burned-out neoplasm, and avulsive cortical irregularities [2]. Similar-looking bone erosions may occasionally be associated with calcific tendinitis, but they are characterized by calcium deposits in the adjacent soft tissues [5,6].

Chronic avulsion injury of the deltoid also can present with similar clinical and imaging characteristics, posing a dilemma for the radiologist. MRI is helpful with features such as prominent thickness and irregularity of the cortex, increased T2-weighted signal intensity in the tendon insertion, and edema in the adjacent soft tissue directing towards avulsive injury [5]. The presence of increased T2-weighted signal intensity in the tendon and surrounding adjacent soft tissue is an indicator for both acute and chronic avulsive injuries of the deltoid tubercle. The absence of such T2 hyperintensity favors the presence of pseudotumor deltoideus.

Neoplasm should be excluded during the evaluation of bone abnormality. Due to their eccentric position, two benign neoplasms, fibrous cortical defects and non-ossifying fibromas, are given special consideration as differentials [7,8]. They differ from pseudotumor deltoideus, in that they occur mostly in immature skeletons, are metaphyseal, and rarely manifest symptoms unless associated with pathologic fracture [7].

In this article, cortical thickening and intracortical lucency were observed at the site of the deltoid insertion. The radiological imaging findings were consistent with both pseudotumor deltoideus and chronic avulsion injury of the deltoid. There was no bony fragment, and MRI revealed no edema in the soft tissue adjacent to the deltoid insertion. The diagnosis of pseudotumor deltoideus was made after considering the patient's age, symptoms, a lack of history of injury from sports or other strenuous activity, and imaging findings. The same was confirmed during a two-month follow-up following physiotherapy, NSAIDs, and conservative care that resulted in symptom remission. Few studies have found a link between arm dominance and the prevalence of pseudotumor deltoideus [1]. In our cases, only one appeared on the dominant arm, implying that there was no relationship with arm dominance.

Various studies such as those of Singh and Tanwar, Morgan et al., and Adiguzel et al. described similar radiological clues of pseudotumor deltoideus [1,2,9]. Symptomatic individuals presented with an area of cortical thickening and intracortical lucency at the site of deltoid attachment detected in both conventional radiography and CT. In addition, MRI reveals T1- and T2-weighted hyperintense signal changes in the cortex with no associated soft tissue and marrow edema. In previous studies, pseudotumor deltoideus in patients, which resembled our symptomatic cases, was seen in their dominant arm [1,2]. The definitive diagnosis of pseudotumor deltoideus is based on clinical and radiographic evidence, particularly when the differentials have been ruled out.

## Conclusions

A diagnosis of pseudotumor deltoideus should be taken into consideration in patients with shoulder pain when imaging reveals cortical thickening of the proximal humerus at the site of the deltoid insertion accompanied with irregularity and intracortical lucency on CT and T2 hyperintensity on MRI without any surrounding soft tissue changes. Unwanted biopsies should be avoided, and MRI is useful in the differential diagnosis.
